# The Limits between Schizophrenia and Bipolar Disorder: What Do Magnetic Resonance Findings Tell Us?

**DOI:** 10.3390/bs12030078

**Published:** 2022-03-15

**Authors:** Mirona Letitia Dobri, Alexandre Paim Diaz, Sudhakar Selvaraj, Joao Quevedo, Consuelo Walss-Bass, Jair C. Soares, Marsal Sanches

**Affiliations:** 1 Center of Excellence on Mood Disorders, Faillace Department of Psychiatry and Behavioral Sciences, McGovern Medical School, The University of Texas Health Science Center at Houston (UTHealth), Houston, TX 77054, USA; alexandre.paimdiaz@uth.tmc.edu (A.P.D.); sudhakar.selvaraj@uth.tmc.edu (S.S.); joao.l.dequevedo@uth.tmc.edu (J.Q.); consuelo.walssbass@uth.tmc.edu (C.W.-B.); jair.c.soares@uth.tmc.edu (J.C.S.); marsal.sanches@uth.tmc.edu (M.S.); 2 Translational Psychiatry Program, Faillace Department of Psychiatry and Behavioral Sciences, McGovern Medical School, The University of Texas Health Science Center at Houston (UTHealth), Houston, TX 77054, USA

**Keywords:** bipolar disorder, schizophrenia, magnetic resonance imaging, neuroimaging, pathophysiology

## Abstract

Schizophrenia and bipolar disorder, two of the most severe psychiatric illnesses, have historically been regarded as dichotomous entities but share many features of the premorbid course, clinical profile, genetic factors and treatment approaches. Studies focusing on neuroimaging findings have received considerable attention, as they plead for an improved understanding of the brain regions involved in the pathophysiology of schizophrenia and bipolar disorder. In this review, we summarize the main magnetic resonance imaging findings in both disorders, aiming at exploring the neuroanatomical and functional similarities and differences between the two. The findings show that gray and white matter structural changes and functional dysconnectivity predominate in the frontal and limbic areas and the frontotemporal circuitry of the brain areas involved in the integration of executive, cognitive and affective functions, commonly affected in both disorders. Available evidence points to a considerable overlap in the affected regions between the two conditions, therefore possibly placing them at opposite ends of a psychosis continuum.

## 1. Introduction

Even though the term “psychosis” encompasses many nosological entities, the current systems utilized for the classification of mental illnesses adopt a categorical approach, based on clinical and phenomenological criteria rather than underlying epidemiological, biological, or genetic factors. This clear demarcation is meant to aid in clinical treatment decisions, make research endeavors reproducible and establish a framework for investigations.

Specifically with regard to schizophrenia (SCZ) and bipolar disorder (BD), this segregated approach is an evolutionary consequence of the Kraepelinian dichotomy coined more than a century ago, which established dementia praecox and manic-depressive insanity as two separate illnesses [1,2]. The term dementia praecox was employed by Kraepelin to define a disease entity characterized by poor cognitive and social functioning, disorganization of thought and behavior, decreased volition, early onset in life and a poor long-term outcome, with less emphasis on positive symptoms such as delusions and hallucinations. Eugen Bleuler, who coined the term “schizophrenia,” did so by associating it with symptoms that would later be adopted as pathognomonic for that disorder, such as splitting of thought or blunted affect. On the other hand, manic-depressive illness, as envisioned by Kraepelin, was described as a cyclical course of mood episodes, with or without mania, followed by intermittent windows of remission and without long-term cognitive deterioration.

The definitions of the *Diagnostic and Statistical Manual of Mental Disorders*, Fifth Edition (DSM-5), however, depart from these constructs. According to DSM-5, the diagnosis of SCZ requires a minimum of one month of at least two elements from a group of symptoms including delusions, hallucinations, disorganized speech, disorganized behavior and negative symptoms, such as affective flattening, with at least one corresponding to delusions, hallucinations or disorganized speech. BD type I is diagnosed with the presence of at least one episode of mania or hypomania regardless of depressive episode history. Mania includes at least one week of elevated, expansive or irritable mood accompanied by three or more of the following symptoms: inflated self-esteem or grandiosity, decreased need for sleep, increased talkativeness, racing thoughts, distractibility, increased goal-directed activity or psychomotor agitation and engagement in activities with the potential for harmful consequences, with a possible association with mood-congruent or incongruent psychotic features [1,3,4,5,6]. Consequently, individuals with BD experiencing psychotic features may be, at times, difficult to differentiate from those with SCZ experiencing acute psychotic symptomatology. It has been proposed that this clinical overlap, in addition to shared findings related to their premorbid course, genetic risk factors and response to pharmacological treatment, supports the existence of a “psychosis continuum”, according to which SCZ and BD would represent the two ends of a spectrum instead of two well-delineated disorders. 

Given the numerous advances observed over the last decades with regard to neuroimaging findings in mental disorders and their pathophysiological significance, we performed a review of the main magnetic resonance imaging (MRI) findings in BD and SCZ, as a point of exploration of these diagnoses as separate entities or as part of a continuum. We conducted a comprehensive literature search on Google Scholar and PubMed for potentially relevant English-language articles regardless of the year of publication. We used combinations of the following keywords: schizophrenia, bipolar disorder, bipolar, imaging, neuroimaging, MRI, magnetic resonance imaging, DTI, diffusion tensor imaging and resting-state fMRI. Articles matching these criteria, based on the title and abstract, were retrieved for a more detailed evaluation. We included studies focusing solely on schizophrenia or solely on bipolar disorder and studies comparing the two. We focused on studies assessing results from brain volumetric studies (structural imaging based on conventional MRI), diffusion-weighted imaging (DWI) and resting-state functional MRI (rs-fMRI) among patients with SCZ and BD. The evidence was critically discussed, with a focus on the categorical versus dimensional specificity of the findings. 

## 2. Neuroimaging Findings in Schizophrenia and Bipolar Disorder

### 2.1. Structural Imaging Based on Conventional MRI Findings

Over the last decades, conventional MRI has fully replaced computerized tomography as the method of choice for examining the structural neuroanatomy of the brain. Nonetheless, studies with conventional MRI display great heterogeneity in terms of image acquisition and analysis, varying between manual tracing approaches such as examining regions of interest (ROI), anteriorly defined anatomically and computational methods such as voxel-based morphometry (VBM), deformation-based morphometry (DBM), surface-based morphometry (SBM) and voxel-based lesion symptom mapping (VLSM), which offer automated analysis of brain differences across the whole brain [7]. These differences seem to be, at least in part, responsible for important variations regarding neuroimaging findings in psychiatric disorders. 

Still, MRI studies have consistently reported cortical and subcortical gray matter abnormalities in both SCZ and BD (Table 1). A meta-analysis encompassing data from 1646 patients diagnosed with SCZ and 1690 healthy controls (HC) indicated extensive gray matter reductions in the frontal, temporal, thalamic and striatal regions in patients with SCZ compared to HC [8]. Another meta-analysis, conducted by the ENIGMA Schizophrenia Working Group, found widespread cortical thinning in all brain regions (except the pericalcarine region), with certain areas (such as the fusiform, parahippocampal and inferior temporal gyri) demonstrating a thinner cortex while others (superior parietal cortex, precuneus and paracentral lobule) displayed a significantly thicker cortex. In addition, earlier age at onset and longer illness duration were associated with a thinner insular cortex. The results also show widespread smaller cortical surface area, but with no regional specificity. Both measures evidenced more prominent results in the frontal and temporal lobes [9]. Moreover, the findings of the ENIGMA consortium point to associations between decreased cortical thickness in SCZ and treatment history, with a larger effect size cortex for first-generation antipsychotics compared to second-generation antipsychotics. Furthermore, a study enabling optimized VBM and resting-state functional connectivity analysis observed gray matter volume decreases in the right side of the anterior cingulate gyrus, middle temporal gyrus and superior temporal gyrus in first-episode patients before treatment initiation, with positive correlations between gray matter volume and functional status [10]. Findings of a progressive volumetric decline were confirmed in a meta-analytic study conducted by Brent et al. focusing on individuals at high genetic risk for SCZ and patients with early onset SCZ, with gray matter volume reductions especially evident in frontotemporal structures [11].

Previous research also described subcortical abnormalities in patients with SCZ. The most consistent finding among the research was that of a smaller hippocampus [12,13], amygdala, thalamus and nucleus accumbens. Decreased intracranial volumes [13,14]; enlarged lateral ventricles [13,14]; and increased putamen and pallidum volume have also been consistently reported [13]. The latter has been found to be positively correlated with duration of illness and seems to be directly associated with medication effects of antipsychotic medication on basal ganglia volumes [13]. In a recent large-scale, multisite study, Okada et al. described increases in both the whole-caudate-nucleus volume and putamen among patients with SCZ [15]. 

Other areas of the brain have been found to display structural alterations in SCZ. A more recent study specifically described gray matter decreases in the bilateral middle orbital gyri, bilateral inferior frontal gyri, left anterior cingulate cortex and left medial frontal gyrus. In the temporal lobe, gray matter volume reductions are reported in the bilateral insular lobes, bilateral olfactory cortices and right hippocampus, left angular gyrus, thalamus and caudate nucleus [16]. 

Moreover, decreased whole-brain volume and lateral ventricular enlargement in patients with SCZ compared with HC [17,18] (with enlargement slightly more prominent in the left lateral ventricle) are considered classic neuroimaging findings in SCZ and seem to be correlated with negative symptomatology [18]. In contrast with previous results, however, van Erp et al. found an inverse association between negative symptom severity and lateral ventricle volume [13]. 

Similarly, findings on the hippocampus vary greatly. In line with previous results showing decreased hippocampal volume in SCZ patients [19], a multicenter study also found a greater decrease in hippocampal volume in patients with a higher mean age of onset. Conversely, in a meta-analysis, Adriano et al. reported similar hippocampal volume reductions in first-episode and chronic patients with SCZ [12]. One study comparing first-episode, unmedicated patients with SCZ and HC associated decreased gray matter hippocampal volumes with cognitive deficits primarily in the areas of working memory and verbal and visual learning [20]. Last, the two large-scale consortium studies also reported decreased amygdala volume in patients with SCZ compared to HC [13,15].

In BD, reviews of earlier findings suggested no significant difference in brain volumes of patients compared to HC [21], although findings are fairly heterogenous among different studies. A later meta-analysis described more specific gray matter decreases in BD subjects compared with HC in the right insula, perigenual anterior cingulate, left insula and subgenual anterior cingulate [22], overlapping substantially with those found in SCZ groups in the same study, while Bora et al. contended that gray matter decreases in the left anterior cingulate cortex and right fronto-insular cortex are the most replicable abnormalities in patients with BD [23]. One study found no differences overall in the prefrontal region volume between patients with BD and HC, although volumetric gray matter decreases were noted in the left superior, middle and right prefrontal subregions [24]. Focusing on more specific regions of the prefrontal cortex, several studies found volume reductions in the subgenual prefrontal cortex of the anterior cingulate [25,26]. The latest literature supports widespread bilateral cortical thinning in BD patients in frontal, temporal and parietal regions [27], with thinning being more pronounced in the left pars opercularis, left fusiform gyrus and left rostral middle frontal cortex. 

The ENIGMA Bipolar Disorder Working Group meta-analysis encompasses MRI data extracted from 6503 cases, 2447 of which have a BD diagnosis. This working group also published results on subcortical abnormalities in bipolar patients [28], which showed decreased bilateral hippocampus volumes, decreased amygdala and thalamus and lateral ventricle enlargement. They also found hippocampal volume to be inversely associated with age in patients with BD. These results contrast with previous data showing no difference in hippocampal volume [19,29] and increased thalamic volume [29] in bipolar patients versus controls.

Several studies also show the consistent finding of increased amygdala volume in bipolar patients [19,30], although a meta-analytic study focusing on amygdala imaging findings in patients with SCZ and BD found that while SCZ patients exhibited decreased amygdalae bilaterally, no differences in amygdala volume were observed among patients with BD [31]. Lithium therapy has also been shown to have a positive impact on amygdala gray matter volume, as a study found larger amygdalae in euthymic bipolar patients on lithium therapy compared to untreated patents [32]. 

Last, similar to the findings among patients with SCZ, enlarged lateral ventricles have been consistently reported in BD [17,29,33]. Interestingly, patients with multiple mood episodes have been shown to exhibit larger ventricular volumes compared to first-episode patients [29].

MRI structural neuroimaging studies provide valuable evidence for the better understanding of the pathophysiology of schizophrenia and bipolar disorders. Nonetheless, the static nature of the findings in question can limit their interpretation and significance, as it allows only for inferences regarding the brain circuit abnormalities involved in these conditions. In the next two sections, we will discuss techniques able to provide a more refined view of brain connectivity in mental illnesses.

### 2.2. Diffusion-Weighted Imaging Findings

The existence of microstructural abnormalities in white matter tracts underlying white matter connectivity dysfunction across the brain has been a hypothesis widely discussed as a potential causative mechanism for psychotic spectrum disorders such as SCZ and BD. DWI includes different imaging techniques but, given the scope of the present work, we limited our literature review to diffusion tensor imaging (DTI), which measures diffusivity of water molecules along white matter tracts and is utilized to assess brain connectivity [34,35]. One of the two most used measurements is the fractional anisotropy (FA), which measures directionality of water along white matter fibers, with low values (close to 0) reflecting disrupted integrity of the axonal myelin sheath. The other is the apparent diffusion coefficient (ADC), where high values imply increased diffusion of water, suggesting decreased organization of tract fibers [35,36]. 

The most consistent and highly replicated DTI findings in patients with SCZ show decreased white matter integrity across the prefrontal and temporal regions and in the fronto-temporal circuits (Table 2) [34,37]. Kubicki et al. described four structures of importance connecting the frontal and temporal lobes: the uncinate fasciculus (UF), the cingulum bundle, the fornix and the arcuate fasciculus, all of which demonstrate abnormalities in SCZ patients compared to HC [34]. In another study, the authors reported decreased FA bilaterally in the anterior and middle portion of the cingulum, superior occipito-frontal fasciculus, internal capsule (IC) and fornix and corpus callosum (CC), as well as in the right inferior occipito-frontal fasciculus and left arcuate fasciculus [38]. There is substantial evidence of white matter abnormalities, measured as decreased FA in the UF [39,40,41] and anterior thalamic radiation (ATR), in patients with SCZ [39,41]. Sussmann et al. more specifically described lower FA in the frontal part of the UF and anterior limb of the internal capsule (ALIC) and inferior fronto-occipital fasciculus [41]. FA was not found to be associated with age, duration of illness, treatment with psychotropic medication or psychotic symptoms in patients with SCZ [39]. Multiple studies document abnormalities at the level of the CC. Li et al. found significantly decreased FA in all regions of the CC (anterior genu, posterior genu, posterior body, anterior splenium) in patients with SCZ compared to HC [42]. In a study using the whole-brain approach followed by an ROI approach, Skudlarski found decreased FA in all 29 regions examined in patients with SCZ [43]. The ENIGMA Schizophrenia Working Group [44] described a widespread decrease in FA across the whole-brain white matter skeleton, with larger effect sizes in the anterior corona radiata, CC (genu and body) and ALIC, followed by the fornix, posterior thalamic radiation, superior fronto-occipital fasciculus, cingulum, superior longitudinal fasciculus, UF, IC and external capsule. 

In contrast, in most studies, patients with BD exhibit abnormalities in the white matter tracts connecting the frontal lobe and especially the prefrontal cortex to other cortical and subcortical regions (Table 2) [45]. Adler et al. also described decreased FA in multiple ROIs located 25 to 30 mm above the anterior commissure. In a review of multiple studies, Heng et al. mentioned decreased FA and increased ADC in the IC, UF and CC [35]. Decreased FA values have been found in the UF and ATR in multiple studies [39,41], along with the left ALIC and superior thalamic radiation [41]. A 2011 study utilizing DTI tractography reported substantially low FA values in the ATR bilaterally and in the left UF [46]. Another tractography study compared euthymic bipolar I patients with HC and described decreased tract length in the genu, body and splenium of the CC and decreased fiber density in the genu and body of the CC and inferior longitudinal fasciculus bilaterally [47], and found no differences in FA measures between bipolar patients and HC, except in one of the corticospinal tracts. In a multicenter tractography study carried out by Sarrazin et al., the results show significantly decreased FA in the anterior segment of the left arcuate fasciculus, body and splenium of the CC and long fibers of the left cingulum. Bipolar patients with psychotic features showed substantially lower FA values in the body of the CC compared with bipolar patients without psychotic features [48]. In line with previous literature findings, the ENIGMA consortium study by Favre et al. showed widespread alterations in white matter microstructure more pronounced in the CC and cingulum. Their results show lower FA in 29 of 44 regions analyzed. Higher FA was found to be associated with a later age of onset, shorter duration illness and lithium therapy [49].

### 2.3. Resting-State Functional MRI

Functional MRI (fMRI) represents another more recently developed modality for studying brain function and allows the acquisition of multiple images from the same patient while offering improved spatial and temporal resolution compared to older imaging techniques. Using the blood-oxygen-level-dependent (BOLD) technique, fMRI measures the difference in concentration between deoxyhemoglobin and oxyhemoglobin that occurs when increased neural activity within a certain brain region leads to an increase in cerebral blood flow [21,29]. While some studies focus on investigating brain activity during the execution of a specific task, more novel approaches investigate brain connectivity during the resting state [50,51], as resting BOLD activity is thought to map the default functional connectivity for the explored regions. Many studies argue the existence of functional connectivity abnormalities as an underlying pathophysiological mechanism in both SCZ and BD [52], predominantly at the level of prefrontal cortical and limbic regions (Table 3) [53]. Therefore, in the present paper, we will focus on resting-state functional MRI findings. Most rs-fMRI studies use the ROI and independent component analysis (ICA) approach, with some measuring the amplitude of low-frequency fluctuations (ALFF) and restricted global connectivity (rGBC) [51].

In one study, Chai et al. evaluated the medial prefrontal cortex (MPFC) as a structure crucial to the pathophysiology of BD and SCZ. This seed-driven approach explores the functional connectivity of a chosen seed region to other regions of the brain [52]. They described an absence of anticorrelated activity between the MPFC and dorsal lateral prefrontal cortex (DLPFC) in SCZ patients, as well as diminished anticorrelated activity between the MPFC and insula and ventral lateral prefrontal cortex (VLPFC). [52] The results of a meta-analysis of fMRI and PET studies on SCZ patients showed hypoactivation in the ventral MPFC, left hippocampus, posterior cingulate cortex and precuneus and hyperactivation of the lingual gyrus bilaterally [54]. 

Another approach to studying resting-state connectivity is through analyzing within-network and between-network connectivity at the level of the five most prominent resting-state networks in the brain: default mode (DMN), fronto-parietal (FP), cingulo-opercular (CO) and cerebellar (CER), which are all believed to modulate cognitive function, and the salience network (SAL). The latter includes parts of the anterior cingulate, prefrontal cortex and insula, and is involved in recruiting brain regions for sensory input processing [55,56]. In one study, patients with SCZ showed decreased connectivity within the CO network compared to HC, but less so in comparison with the BD group. Decreased FP-CO and FP-CER connectivity was also found in SCZ patients, along with decreased connectivity between SAL and CER, found only in SCZ patients [55]. Another study focusing on functional connectivity between different neural network pairs found decreased connections between the fronto-occipital network and the DMN/prefrontal network [56]. In this study, using ICA, Meda et al. found affected connections between fronto-premotor and meso/paralimbic networks among patients with SCZ [56]. This data-driven approach decomposes the brain into independent components, each shown as a functional map computed from data patterns [51,52,56]. Another study comparing SCZ patients to bipolar patients and controls showed decreased global connectivity for SCZ patients, with intermediate values for bipolar patients compared to controls. Additionally, both groups of patients had lower values in the paracingulate gyrus and right thalamus. SCZ patients had significantly lower connectivity in the temporal occipital fusiform cortex, left caudate nucleus and left thalamus [57]. 

Chai et al., focusing on MPFC, described no anticorrelation between MPFC and DLPFC in patients with BD compared to HC, but positive correlations or hyperconnectivity between MPFC-insula and MPFC-VLPFC relationships were found only in BD [52]. In a study assessing resting-state brain activity in bipolar depression, Liu et al. quantified the ALFF in the fMRI signal. The ALFF measures spontaneous neural activity of the BOLD signal. They reported increased ALFF in the left insula, right caudate nucleus, bilateral temporal gyrus and bilateral inferior frontal gyrus and decreased ALFF measures in the left postcentral gyrus, left parahippocampal gyrus and cerebellum in bipolar depression patients compared to the control group [58]. In the study analyzing connectivity among the five main neural networks in the brain, bipolar patients have shown decreased within-network connectivity in the CO and decreased connectivity between CO and CER. A decreased connectivity between SAL and CO networks only in bipolar patients was also reported [55]. Meda et al. found an affected connection between the fronto-occipital network and the DMN/prefrontal network in bipolar patients, a characteristic shared by SCZ patients but to a lesser extent. Additionally, the network consisting of the meso/paralimbic regions (mesial temporal cortex, amygdala, para-hippocampus, hippocampus) showed significantly increased connectivity with other mood-regulatory regions (subgenual cingulate, ventrolateral prefrontal cortex, orbitofrontal cortex, insula) only in bipolar patients. This network pair is thought to be involved in emotional expression and regulation [56]. In a study comparing unmedicated bipolar patients with major depressive disorder patients and controls, Anand et al. used the ROI approach to measure BOLD fluctuations and found similar decreases in corticolimbic connectivity compared to the healthy subject group but noted decreased connectivity between the pregenual anterior cingulate cortex and the left amygdala only in BD [59]. Connectivity abnormalities between the prefrontal and anterior cingulate cortices and the mesolimbic areas, including the thalamus, amygdala and insula, were reported by Vargas et al. in their systematic review of all bipolar patients, regardless of state, compared to control groups [51]. In a study employing the rGBC method and focusing on prefrontal cortex functional connectivity within-region and to other regions in bipolar I disorder, the results show reduced MPFC within-region connectivity in bipolar patients with a psychosis history compared to those without psychosis and the control group. However, there was hyperconnectivity to the amygdala and decreased connectivity for amygdala-DLPFC networks in BD, which was more prominent in those exhibiting psychotic features. This method measures connectivity between a voxel and every other voxel within a restricted region [60]. 

## 3. Discussion

In this review, we aimed at summarizing the main MRI findings described in SCZ and BD, while critically analyzing these findings with regard to the “categorical versus continuum” approach to those conditions.

In SCZ, overall gray matter volume reductions with concomitant ventricle enlargement seem to be a consistent finding. Gray matter structural abnormalities predominant in SCZ primarily involve the frontal, temporal, thalamic and striatal areas. The affected regions are consistent throughout the literature, with an emphasis on volume decreases in the anterior cingulate cortex, inferior frontal gyrus, middle frontal gyrus, insula, hippocampus, amygdala and thalamus and an observed increase in basal ganglia volumes [13]. White matter connectivity dysfunctions in SCZ are in alignment with the gray matter alterations and primarily involve the frontotemporal circuitry. Functional connectivity studies are heterogeneous in methodology, but results revolve around connectivity abnormalities in the prefrontal and limbic regions in SCZ. Additionally, patients with SCZ display affected connectivity between the fronto-occipital network and the DMN/prefrontal network and between the fronto-premotor and meso/paralimbic networks. These disrupted connections likely affect multiple aspects, including emotional processing, cognitive control and affect.

On the other hand, structural imaging findings in BD show greater variability, although seem to be concentrated on gray matter volume decreases, predominantly in the frontal and prefrontal areas, along with the temporal and parietal areas. The regions most commonly involved are the anterior cingulate cortex, especially the perigenual and subgenual prefrontal parts and the insular lobes. Lateral ventricle enlargement might correspond to a compensatory feature that often accompanies cortical thinning in BD patients. Similarly, subcortical structures show heterogeneous results in bipolar patients, with inconsistent data regarding amygdala, thalamic and hippocampal volumes. Structural connectivity studies report alterations in the white matter fibers connecting the prefrontal cortex to other cortical and subcortical regions. Abnormalities have been found at the level of the IC, UF, left ALIC, anterior and superior thalamic radiations, cingulum bundle, arcuate fasciculus and CC, with variability in the CC regions affected. The main functional connectivity findings in BD focus on hyperconnectivity between MPFC-insula and MPFC-VLPFC, but no anticorrelated activity between MPFC and DLPFC. Additionally, increased ALFF has been measured in the left insula, caudate nucleus, bilateral temporal gyrus and bilateral inferior frontal gyrus. These regions are part of the prefrontal-limbic system and the associated striatal system, which are involved in cognition and emotional processing. Bipolar patients also display decreased connectivity in the CO network and between the CO and the CER. Moreover, decreased connectivity between CO and SAL seems to be particularly associated with BD. Patients with BD exhibit altered connectivity between the fronto-occipital network and the DMN/prefrontal network, as well as increased connectivity between the meso/paralimbic regions and the subgenual cingulate, VLPFC, orbitofrontal cortex and insula. These findings suggest reduced functional integration within the prefrontal cortex as well as between the prefrontal and limbic areas, areas involved in the regulation of emotions.

Therefore, in addition to phenotypical overlap, SCZ and BD seem to share several findings with regard to structural and functional MRI findings. Both conditions show regional decreases in gray matter content, although a widespread decrease in cortical gray matter volume seems to be more consistently associated with SCZ than with BD. Decreased gray matter volume centers around areas belonging to the frontal and temporal lobe regions are also found in both conditions. Increases in lateral ventricle volume have been described in both conditions, although patients with SCZ seem to display greater ventricular volumes compared to those with BD. These differences might be related to variations in the timing or intensity of similar pathophysiological processes, which could be the result of a similar disease process with a different intensity [61]. Similarly, the anterior cingulate cortex shows alterations in both conditions. This area is implicated in reward circuits and emotional processing, pathways found to be affected in both disorders. Additionally, the insular cortex, involved in affective information integration and fear mediation, shows similar volume decreases in both conditions [22]. Overall, most alterations evidenced in both SCZ and BD do not appear to have diagnostic specificity, as both diagnoses display a great degree of overlap in the affected regions. Cross-diagnostic studies find a greater magnitude of findings in SCZ compared to BD, which may be a result of neurodegenerative processes involved in the former.

Similarly, despite variations across different studies, decreased hippocampal volume has been found in both conditions, although decreases seem to be slightly more pronounced in SCZ compared to BD. Hippocampal atrophy is thought to be associated with episodic memory impairment; thus, these differences might account for the more significant memory impairments found in patients with SCZ. Moreover, despite heterogeneous findings on the amygdala volume in BD, significantly smaller amygdalae have been found in SCZ compared to BD patients in cross-diagnostic studies. These findings could be attributed to confounding effects of medication, such as larger amygdalae in lithium-treated patients and gray matter loss in patients receiving antipsychotic medication.

Furthermore, DTI studies support the hypothesis of abnormal fronto-thalamic-striatal connectivity as one of the underlying pathological mechanisms in SCZ and BD. Structural connectivity studies also highlight abnormalities in fibers connecting the amygdala with other structures, such as the UF, which connects the amygdala to the orbitofrontal cortex and anterior temporal lobe. This structure, involved in emotion regulation, decision making and episodic memory [34], exhibits decreased FA in both SCZ and BD. The cingulum bundle, with a role in attention, emotional processing, spatial orientation and memory [34], connects parts of the anterior cingulate cortex, dorsomedial frontal cortex and medial temporal lobe to the amygdala, is also jointly affected. Altered FA values have been reported in the genu, body and splenium of the CC in both disorders, which is of particular importance considering the central role of the CC in cognitive processing through interhemispheric informational integration [42]. Most studies also agree on decreased FA values in the arcuate fasciculus, a fiber tract connecting Broca’s and Wernicke’s areas [34], thus being a central instrument in language processing in the brain. Decreased FA values in SCZ and BD may be linked to the presence of auditory hallucinations and language deficits. 

Last, most functional imaging studies support the hypothesis of a common pattern of connectivity dysfunction centering on the frontal and limbic areas in SCZ and BD. As such, the MPFC is considered a relay station in the prefrontal cortex modulation of the limbic structures, thus leading to emotional dysregulation in BD and inefficient cognitive processing in SCZ [52]. The decoupling in the MPFC and DLPFC seen in both conditions is consistent with the neuropsychological deficits observed in both. In contrast, the finding of increased connectivity between the MPFC, VLPFC and insula, which seems to be present mostly in BD, could be attributed to the role of these circuits in emotional processing. In consonance with this hypothesis, decreased connectivity within the CO network was found in both diseases but seems to be less pronounced in SCZ compared to BD, suggesting that the CO network may have a more prominent role in emotion regulation than in cognition.

Methodological factors may explain some of the inconsistent findings regarding neuroimaging findings in both conditions. Differences in phenotypical characterization, especially in the case of BD, as well as distinct illness trajectories and treatment histories, at times limit the comparability and generalizability of MRI studies. The same applies to technical factors such as strength of the MRI scanner and the chosen approach for imaging analysis. Therefore, the findings included in the present review, as well their interpretation from a pathophysiological standpoint, need to be treated with caution. 

## 4. Conclusions

This review provides evidence of coinciding anatomical and functional brain changes in SCZ and BD that could account for the substantial overlap in phenomenology and the consequent similarity in pharmacological treatment strategies. Taken together, the neuroimaging studies examined show disruptions in gray matter structure, white matter tract integrity and functional connectivity in both diseases, predominantly in the prefrontal and limbic systems and connecting circuitry. Although there are no significant differences across regions between conditions, there appears to be a greater degree of dysfunction in SCZ compared to BD. These findings challenge the Kraepelinian dichotomic classification and support the hypothesis that both diseases belong to the same continuum. 

## Figures and Tables

**Table 1:** Gray matter volume abnormalities in schizophrenia and bipolar disorder.

Structure	Schizophrenia	Bipolar Disorder
Whole-brain volume	↓↓	↓
Fusiform gyrus	↓	↓
Parahippocampal gyrus	↓	↓
Inferior temporal gyrus	↓	Normal
Middle temporal gyrus	↓	↓
Superior temporal gyrus	↓	↓
Hippocampus	↓↓	↓
Amygdala	↓↓	↓ or ↑
Insula	↓	↓
Fronto-insular cortex	Normal or ↓	↓
Anterior cingulate cortex	↓	↓
Inferior frontal gyrus	↓	↓
Medial frontal gyrus	↓	↓
Middle orbital gyrus	↓	Normal
Olfactory cortex	↓	Normal
Rostral middle frontal cortex	↓	↓
Thalamus	↓	↓ or ↑
Nucleus accumbens	↓	Normal
Putamen	↑	Normal
Pallidum	↑	Normal
Lateral ventricle	↑↑	↑

↓ = decreased volume; ↑ = increased volume; ↓↓ = relatively greater decrease in volume; ↑↑ = relatively greater increase in volume.

**Table 2:** Fractional anisotropy changes in schizophrenia and bipolar disorder.

Structure	Schizophrenia	Bipolar Disorder
Uncinate fasciculus	↓	↓
Arcuate fasciculus	↓	↓
Superior occipito-frontal fasciculus	↓	Normal
Inferior occipito-frontal fasciculus	↓	↓
Superior longitudinal fasciculus	↓	↓
Inferior longitudinal fasciculus	↓	↓
Anterior thalamic radiation	↓	↓
Posterior thalamic radiation	↓	↓
Cingulum bundle	↓	↓↓
Corpus callosum	↓↓	↓↓
Fornix	↓	Normal or ↓
Corona radiata	↓↓	↓
Internal capsule	↓	↓
Anterior limb of internal capsule	↓↓	↓
External capsule	↓	Normal or ↓

↓ = decreased FA; ↓↓ = relatively greater decrease in FA.

**Table 3:** Functional abnormalities in schizophrenia and bipolar disorder.

Structures	Schizophrenia	Bipolar Disorder
MPFC	↓	↓
MPFC-DLPFC	↓↓	↓↓
MPFC-insula	↓	↑
MPFC-VLPFC	↓	↑
VMPFC	↓	Normal
Hippocampus	↓	Normal
Lingual gyrus	↑	Normal
Paracingulate gyrus	↓	↓
Thalamus	↓	↓
CO	↓	↓↓
FP-CO	↓	Normal
CO-CER	Normal	↓
FP-CER	↓	Normal
SAL-CER	↓	Normal
SAL-CO	Normal	↓
Fronto-occipital—DMN/prefrontal	↓	↓
Fronto-premotor—meso/paralimbic	↓	Normal
Meso/paralimbic—VLPFC	Normal	↓
Meso/paralimbic—insula	Normal	↓
Global connectivity	↓↓	↓

↓ = decreased functional connectivity; ↑ = increased functional connectivity; ↓↓ = relatively greater decrease in functional connectivity.

## Data Availability

Not applicable.

## Abbreviations

ADC	apparent diffusion coefficient
ALFF	amplitude of low-frequency fluctuations
ALIC	anterior limb of internal capsule
ATR	anterior thalamic radiation
BD	bipolar disorder
BOLD	Blood-oxygen-level-dependent
CC	corpus callosum
CER	cerebellar network
CO	cingulo-opercular network
DLPFC	dorsal lateral prefrontal cortex
DMN	default mode network
DTI	diffusion tensor imaging
DWI	diffusion-weighted Imaging
FA	fractional anisotropy
FP	fronto-parietal network
fMRI	functional magnetic resonance imaging
HC	healthy controls
IC	internal capsule
ICA	independent component analysis
MRI	magnetic resonance imaging
MPFC	medial prefrontal cortex
rGBC	restricted global connectivity
ROI	regions of interest
rs-fMRI	resting-state functional magnetic resonance imaging
SAL	salience network
SCZ	schizophrenia
UF	uncinate fasciculus
VBM	voxel-based morphometry
VLPFC	ventral lateral prefrontal cortex

## References

[1] Pearlson G.D. (2015). Etiologic, phenomenologic, and endophenotypic overlap of schizophrenia and bipolar disorder. Annu. Rev. Clin. Psychol..

[2] DeRosse P., Karlsgodt K.H. (2015). Examining the Psychosis Continuum. Curr. Behav. Neurosci. Rep..

[3] Esterberg M.L., Compton M.T. (2009). The psychosis continuum and categorical versus dimensional diagnostic approaches. Curr. Psychiatry Rep..

[4] Fischer B.A., Carpenter W.T. (2009). Will the Kraepelinian dichotomy survive DSM-V?. Neuropsychopharmacology.

[5] van Rooijen G., Isvoranu A.M., Meijer C.J., van Borkulo C.D., Ruhe H.G., de Haan L., Investigators G. (2017). A symptom network structure of the psychosis spectrum. Schizophr. Res..

[6] American Psychiatric Association (2013). Diagnostic and Statistical Manual of Mental Disorders.

[7] Perlini C., Bellani M., Brambilla P. (2012). Structural imaging techniques in schizophrenia. Acta Psychiatr. Scand..

[8] Fornito A., Yucel M., Patti J., Wood S.J., Pantelis C. (2009). Mapping grey matter reductions in schizophrenia: An anatomical likelihood estimation analysis of voxel-based morphometry studies. Schizophr. Res..

[9] van Erp T.G.M., Walton E., Hibar D.P., Schmaal L., Jiang W., Glahn D.C., Pearlson G.D., Yao N., Fukunaga M., Hashimoto R. (2018). Cortical Brain Abnormalities in 4474 Individuals With Schizophrenia and 5098 Control Subjects via the Enhancing Neuro Imaging Genetics Through Meta Analysis (ENIGMA) Consortium. Biol. Psychiatry.

[10] Lui S., Deng W., Huang X., Jiang L., Ma X., Chen H., Zhang T., Li X., Li D., Zou L. (2009). Article association of cerebral deficits with clinical symptoms in antipsychotic-naive first-episode schizophrenia: An optimized voxel-based morphometry and resting state functional connectivity study. Am. J. Psychiatry.

[11] Brent B.K., Thermenos H.W., Keshavan M.S., Seidman L.J. (2013). Gray matter alterations in schizophrenia high-risk youth and early-onset schizophrenia: A review of structural MRI findings. Child Adolesc. Psychiatr. Clin. N. Am..

[12] Adriano F., Caltagirone C., Spalletta G. (2012). Hippocampal volume reduction in first-episode and chronic schizophrenia: A review and meta-analysis. Neuroscientist.

[13] van Erp T.G., Hibar D.P., Rasmussen J.M., Glahn D.C., Pearlson G.D., Andreassen O.A., Agartz I., Westlye L.T., Haukvik U.K., Dale A.M. (2016). Subcortical brain volume abnormalities in 2028 individuals with schizophrenia and 2540 healthy controls via the ENIGMA consortium. Mol. Psychiatry.

[14] Rimol L.M., Hartberg C.B., Nesvag R., Fennema-Notestine C., Hagler D.J., Pung C.J., Jennings R.G., Haukvik U.K., Lange E., Nakstad P.H. (2010). Cortical thickness and subcortical volumes in schizophrenia and bipolar disorder. Biol. Psychiatry.

[15] Okada N., Fukunaga M., Yamashita F., Koshiyama D., Yamamori H., Ohi K., Yasuda Y., Fujimoto M., Watanabe Y., Yahata N. (2016). Abnormal asymmetries in subcortical brain volume in schizophrenia. Mol. Psychiatry.

[16] Lee D.K., Lee H., Park K., Joh E., Kim C.E., Ryu S. (2020). Common gray and white matter abnormalities in schizophrenia and bipolar disorder. PLoS ONE.

[17] Pearlson G.D., Barta P.E., Powers R.E., Menon R.R., Richards S.S., Aylward E.H., Federman E.B., Chase G.A., Petty R.G., Tien A.Y. (1997). Medial and superior temporal gyral volumes and cerebral asymmetry in schizophrenia versus bipolar disorder. Biol. Psychiatry.

[18] Wright I.C., Rabe-Hesketh S., Woodruff P.W., David A.S., Murray R.M., Bullmore E.T. (2000). Meta-analysis of regional brain volumes in schizophrenia. Am. J. Psychiatry.

[19] Altshuler L.L., Bartzokis G., Grieder T., Curran J., Jimenez T., Leight K., Wilkins J., Gerner R., Mintz J. (2000). An MRI study of temporal lobe structures in men with bipolar disorder or schizophrenia. Biol. Psychiatry.

[20] Guo X., Li J., Wang J., Fan X., Hu M., Shen Y., Chen H., Zhao J. (2014). Hippocampal and orbital inferior frontal gray matter volume abnormalities and cognitive deficit in treatment-naive, first-episode patients with schizophrenia. Schizophr. Res..

[21] Strakowski S.M., DelBello M.P., Adler C., Cecil K.M., Sax K.W. (2000). Neuroimaging in bipolar disorder. Bipolar Disord..

[22] Ellison-Wright I., Bullmore E. (2010). Anatomy of bipolar disorder and schizophrenia: A meta-analysis. Schizophr. Res..

[23] Bora E., Fornito A., Yucel M., Pantelis C. (2010). Voxelwise meta-analysis of gray matter abnormalities in bipolar disorder. Biol. Psychiatry.

[24] López-Larson M.P., DelBello M.P., Zimmerman M.E., Schwiers M.L., Strakowski S.M. (2002). Regional prefrontal gray and white matter abnormalities in bipolar disorder. Biol. Psychiatry.

[25] Drevets W.C., Price J.L., Simpson J.R., Todd R.D., Reich T., Vannier M., Raichle M.E. (1997). Subgenual prefrontal cortex abnormalities in mood disorders. Nature.

[26] Sax K.W., Strakowski S.M., Zimmerman M.E., DelBello M.P., Keck P.E.J., Hawkins J.M. (1999). Frontosubcortical neuroanatomy and the continuous performance test in mania. Am. J. Psychiatry.

[27] Hibar D.P., Westlye L.T., Doan N.T., Jahanshad N., Cheung J.W., Ching C.R.K., Versace A., Bilderbeck A.C., Uhlmann A., Mwangi B. (2018). Cortical abnormalities in bipolar disorder: An MRI analysis of 6503 individuals from the ENIGMA Bipolar Disorder Working Group. Mol. Psychiatry.

[28] Hibar D.P., Westlye L.T., van Erp T.G., Rasmussen J., Leonardo C.D., Faskowitz J., Haukvik U.K., Hartberg C.B., Doan N.T., Agartz I. (2016). Subcortical volumetric abnormalities in bipolar disorder. Mol. Psychiatry.

[29] Strakowski S.M., Delbello M.P., Adler C.M. (2005). The functional neuroanatomy of bipolar disorder: A review of neuroimaging findings. Mol. Psychiatry.

[30] Strakowski S.M., DelBello M.P., Sax K.W., Zimmerman M.E., Shear P.K., Hawkins J.M., Larson E.R. (1999). Brain magnetic resonance imaging of structural abnormalities in bipolar disorder. Arch. Gen. Psychiatry.

[31] Ho N.F., Li Hui Chong P., Lee D.R., Chew Q.H., Chen G., Sim K. (2019). The Amygdala in Schizophrenia and Bipolar Disorder: A Synthesis of Structural MRI, Diffusion Tensor Imaging, and Resting-State Functional Connectivity Findings. Harv. Rev. Psychiatry.

[32] Lopez-Jaramillo C., Vargas C., Diaz-Zuluaga A.M., Palacio J.D., Castrillon G., Bearden C., Vieta E. (2017). Increased hippocampal, thalamus and amygdala volume in long-term lithium-treated bipolar I disorder patients compared with unmedicated patients and healthy subjects. Bipolar Disord..

[33] Soares J.C., Mann J.J. (1997). The anatomy of mood disorders--review of structural neuroimaging studies. Biol. Psychiatry.

[34] Kubicki M., Westin C.F., McCarley R.W., Shenton M.E. (2005). The application of DTI to investigate white matter abnormalities in schizophrenia. Ann. N. Y. Acad. Sci..

[35] Heng S., Song A.W., Sim K. (2010). White matter abnormalities in bipolar disorder: Insights from diffusion tensor imaging studies. J. Neural. Transm..

[36] Bellani M., Yeh P.H., Tansella M., Balestrieri M., Soares J.C., Brambilla P. (2009). DTI studies of corpus callosum in bipolar disorder. Biochem. Soc. Trans..

[37] Keshavan M.S., Collin G., Guimond S., Kelly S., Prasad K.M., Lizano P. (2020). Neuroimaging in Schizophrenia. Neuroimaging Clin. N. Am..

[38] Kubicki M., Park H., Westin C.F., Nestor P.G., Mulkern R.V., Maier S.E., Niznikiewicz M., Connor E.E., Levitt J.J., Frumin M. (2005). DTI and MTR abnormalities in schizophrenia: Analysis of white matter integrity. Neuroimage.

[39] McIntosh A.M., Munoz Maniega S., Lymer G.K., McKirdy J., Hall J., Sussmann J.E., Bastin M.E., Clayden J.D., Johnstone E.C., Lawrie S.M. (2008). White matter tractography in bipolar disorder and schizophrenia. Biol. Psychiatry.

[40] Price G., Cercignani M., Parker G.J., Altmann D.R., Barnes T.R., Barker G.J., Joyce E.M., Ron M.A. (2008). White matter tracts in first-episode psychosis: A DTI tractography study of the uncinate fasciculus. Neuroimage.

[41] Sussmann J.E., Lymer G.K., McKirdy J., Moorhead T.W., Muñoz Maniega S., Job D., Hall J., Bastin M.E., Johnstone E.C., Lawrie S.M. (2009). White matter abnormalities in bipolar disorder and schizophrenia detected using diffusion tensor magnetic resonance imaging. Bipolar Disord..

[42] Li J., Kale Edmiston E., Chen K., Tang Y., Ouyang X., Jiang Y., Fan G., Ren L., Liu J., Zhou Y. (2014). A comparative diffusion tensor imaging study of corpus callosum subregion integrity in bipolar disorder and schizophrenia. Psychiatry Res..

[43] Skudlarski P., Schretlen D.J., Thaker G.K., Stevens M.C., Keshavan M.S., Sweeney J.A., Tamminga C.A., Clementz B.A., O’Neil K., Pearlson G.D. (2013). Diffusion tensor imaging white matter endophenotypes in patients with schizophrenia or psychotic bipolar disorder and their relatives. Am. J. Psychiatry.

[44] Kelly S., Jahanshad N., Zalesky A., Kochunov P., Agartz I., Alloza C., Andreassen O.A., Arango C., Banaj N., Bouix S. (2018). Widespread white matter microstructural differences in schizophrenia across 4322 individuals: Results from the ENIGMA Schizophrenia DTI Working Group. Mol. Psychiatry.

[45] Adler C.M., Holland S.K., Schmithorst V., Wilke M., Weiss K.L., Pan H., Strakowski S.M. (2004). Abnormal frontal white matter tracts in bipolar disorder: A diffusion tensor imaging study. Bipolar Disord..

[46] Lin F., Weng S., Xie B., Wu G., Lei H. (2011). Abnormal frontal cortex white matter connections in bipolar disorder: A DTI tractography study. J. Affect. Disord..

[47] Torgerson C.M., Irimia A., Leow A.D., Bartzokis G., Moody T.D., Jennings R.G., Alger J.R., Van Horn J.D., Altshuler L.L. (2013). DTI tractography and white matter fiber tract characteristics in euthymic bipolar I patients and healthy control subjects. Brain Imaging Behav..

[48] Sarrazin S., Poupon C., Linke J., Wessa M., Phillips M., Delavest M., Versace A., Almeida J., Guevara P., Duclap D. (2014). A multicenter tractography study of deep white matter tracts in bipolar I disorder: Psychotic features and interhemispheric disconnectivity. JAMA Psychiatry.

[49] Favre P., Pauling M., Stout J., Hozer F., Sarrazin S., Abe C., Alda M., Alloza C., Alonso-Lana S., Andreassen O.A. (2019). Widespread white matter microstructural abnormalities in bipolar disorder: Evidence from mega- and meta-analyses across 3033 individuals. Neuropsychopharmacology.

[50] Sanches M., Soares J.C., Soares J.C., Young A.H. (2016). Brain imaging abnormalities in bipolar disorder. Bipolar Disorders: Basic Mechanisms and Therapeutic Implications.

[51] Vargas C., Lopez-Jaramillo C., Vieta E. (2013). A systematic literature review of resting state network—Functional MRI in bipolar disorder. J. Affect. Disord..

[52] Chai X.J., Whitfield-Gabrieli S., Shinn A.K., Gabrieli J.D., Nieto Castanon A., McCarthy J.M., Cohen B.M., Ongur D. (2011). Abnormal medial prefrontal cortex resting-state connectivity in bipolar disorder and schizophrenia. Neuropsychopharmacology.

[53] Chen C.H., Suckling J., Lennox B.R., Ooi C., Bullmore E.T. (2011). A quantitative meta-analysis of fMRI studies in bipolar disorder. Bipolar Disord..

[54] Kuhn S., Gallinat J. (2013). Resting-state brain activity in schizophrenia and major depression: A quantitative meta-analysis. Schizophr. Bull..

[55] Mamah D., Barch D.M., Repovs G. (2013). Resting state functional connectivity of five neural networks in bipolar disorder and schizophrenia. J. Affect. Disord..

[56] Meda S.A., Gill A., Stevens M.C., Lorenzoni R.P., Glahn D.C., Calhoun V.D., Sweeney J.A., Tamminga C.A., Keshavan M.S., Thaker G. (2012). Differences in resting-state functional magnetic resonance imaging functional network connectivity between schizophrenia and psychotic bipolar probands and their unaffected first-degree relatives. Biol. Psychiatry.

[57] Argyelan M., Ikuta T., DeRosse P., Braga R.J., Burdick K.E., John M., Kingsley P.B., Malhotra A.K., Szeszko P.R. (2014). Resting-state fMRI connectivity impairment in schizophrenia and bipolar disorder. Schizophr. Bull..

[58] Liu C.H., Li F., Li S.F., Wang Y.J., Tie C.L., Wu H.Y., Zhou Z., Zhang D., Dong J., Yang Z. (2012). Abnormal baseline brain activity in bipolar depression: A resting state functional magnetic resonance imaging study. Psychiatry Res..

[59] Anand A., Li Y., Wang Y., Lowe M.J., Dzemidzic M. (2009). Resting state corticolimbic connectivity abnormalities in unmedicated bipolar disorder and unipolar depression. Psychiatry Res..

[60] Anticevic A., Brumbaugh M.S., Winkler A.M., Lombardo L.E., Barrett J., Corlett P.R., Kober H., Gruber J., Repovs G., Cole M.W. (2013). Global prefrontal and fronto-amygdala dysconnectivity in bipolar I disorder with psychosis history. Biol. Psychiatry.

[61] Arnone D., Cavanagh J., Gerber D., Lawrie S.M., Ebmeier K.P., McIntosh A.M. (2009). Magnetic resonance imaging studies in bipolar disorder and schizophrenia: Meta-analysis. Br. J. Psychiatry.

